# Transmission of Chikungunya Virus in the Continental United States — Florida, 2014

**Published:** 2014-12-05

**Authors:** Katherine Kendrick, Danielle Stanek, Carina Blackmore

**Affiliations:** 1 Florida Department of Health

On June 27, 2014, the Florida Department of Health in Miami-Dade County was notified by the Florida Poison Information Center Network of a patient with travel to Southeast Asia who was suspected of having chikungunya virus infection. After further investigation and additional testing, it was determined that the patient had not recently traveled to an endemic area, and this case was confirmed as the first locally acquired chikungunya case in the continental United States. Since the first case of locally acquired chikungunya virus infection in the Americas was reported on the Caribbean island of St. Martin in December 2013, the United States has seen an increase in chikungunya cases among travelers returning from areas where chikungunya has become endemic, particularly the Caribbean and South America (1). Compared with other states, Florida has seen an especially large number of chikungunya fever cases. During January 1–October 14, 2014, a total of 272 imported cases were reported in Florida, compared with 1,110 reported in the other 47 contiguous states. In addition, 11 locally acquired chikungunya cases have been identified. The recent spread of the virus and the presence of competent mosquito vectors provide the conditions for transmission of chikungunya virus in Florida (2,3).

Beginning with the first report on June 27, a total of 11 autochthonous chikungunya disease cases in Florida have been reported from four counties: two in Miami-Dade, four in Palm Beach, four in St. Lucie, and one in Broward. All four counties are in South Florida, and three of them (Miami-Dade, Palm Beach, and Broward counties) have reported 131 (48%) of the 272 imported cases in Florida. All 11 locally acquired cases were laboratory-confirmed, seven by polymerase chain reaction. Two of the patients in St. Lucie County live within 1,500 feet (457 meters) of each other, and the cases appear to be linked because of their proximity in space and time; the source is unknown. Of the persons with locally acquired cases, eight (73%) of 11 were female, eight (80%) of 10 were white, and nine (90%) of 10 were non-Hispanic. Median age of the patients was 43 years (range = 29–78 years).

In comparison, of the 272 persons with imported cases, 155 (57%) of 272 were female, 113 (42%) of 267 were white, and 141 (53%) of 265 were non-Hispanic; median age was 48 years (range = 0–88 years). Among imported cases, the most common country of exposure was Haiti (38%), followed by the Dominican Republic (30%); the most common reason for travel was to visit friends and relatives (72%).

Surveillance related to local introductions of chikungunya virus included 50–100 meter cluster investigations around a patient’s residence, enhanced syndromic surveillance, and medical record review. Awareness was increased through media coverage, reverse 911 dialing, and targeted mailings. For more than half of the cases, both locally acquired and imported, local mosquito control workers were notified and deployed to patients’ residences before or on the same day the counties received the positive laboratory test results.

Based on U.S. experiences with dengue virus, which shares the same vectors as chikungunya virus, awareness of the situation in Florida can help inform surveillance activities and control efforts throughout the United States.

Because no vaccine exists to prevent chikungunya fever, the mainstay of prevention is avoiding bites of the mosquitoes that transmit the virus, mostly during daylight hours. The Florida Department of Health and CDC recommend using air conditioning or screens to keep mosquitoes outside, emptying standing water from containers such as flowerpots and buckets where mosquitoes might breed, wearing long-sleeved shirts and long pants, and using insect repellents (1).
